# Biomechanical effect of a lateral hinge fracture for a medial opening wedge high tibial osteotomy: finite element study

**DOI:** 10.1186/s13018-020-01597-7

**Published:** 2020-02-21

**Authors:** Kyoung-Tak Kang, Yong-Gon Koh, Jin-Ah Lee, Jae Jung Lee, Sae Kwang Kwon

**Affiliations:** 1 Department of Mechanical Engineering, Yonsei University, 50 Yonsei-ro, Seodaemun-gu, Seoul, 03722 Republic of Korea; 2 Joint Reconstruction Center, Department of Orthopaedic Surgery, Yonsei Sarang Hospital, 10 Hyoryeong-ro, Seocho-gu, Seoul, 06698 Republic of Korea

**Keywords:** High tibial osteotomy, Lateral hinge fracture, Finite element analysis

## Abstract

**Background:**

This study aimed to investigate the biomechanical effect on the Takeuchi classification of lateral hinge fracture (LHF) after an opening wedge high tibial osteotomy (HTO).

**Methods:**

We performed an FE simulation for type I, type II, and type III in accordance with the Takeuchi classification. The stresses on the bone and plate, wedge micromotion, and forces on ligaments were evaluated to investigate stress-shielding effect, plate stability, and biomechanical change, respectively, in three different types of LHF HTO and with the HTO without LHF model (non-LHF) models.

**Results:**

The greatest stress-shielding effect and wedge micromotion were observed in type II LHF (distal portion fracture). The type II and type III (lateral plateau fracture) models exhibited a reduction in ACL force and an increase in PCL force compared with the HTO without LHF model. However, the type I (osteotomy line fracture) and HTO without LHF models did not exhibit a significant biomechanical effect. This study demonstrates that Takeuchi type II and type III LHF models provide unstable structures compared with the type I and HTO without LHF models.

**Conclusions:**

HTO should be performed while considering a medial opening wedge HTO to avoid a type II and type III LHF as a potential complication.

## Introduction

Medial opening wedge high tibial osteotomy (HTO) is a common treatment for younger and active older patients with medial compartment osteoarthritis and varus malalignment in the knee joint [1]. This procedure is increasingly used because of its benefits for closing wedge osteotomy, such as achieving more predictable correction, maintaining bone stock, and avoiding osteotomy of the fibula, which may compromise the peroneal nerve; however, it may be associated with delayed unions and nonunions [2–5]. Previous studies have investigated the risk for nonunion using this approach, while a few more recent studies have suggested that the risk for nonunion in the opening wedge HTO does not exceed that in the closed wedge technique [6, 7]. Factors that may lead to problems in bone healing include the loss of correction resulting from hardware failure and lateral hinge fracture (LHF) [2, 8].

To perform a successful medial opening wedge HTO, it is necessary to maintain the lateral hinge to provide a fulcrum during the osteotomy, aiming lateral to the upper-third of the proximal tibiofibular joint [9, 10]. However, HTO involves a risk for LHF that may be caused by a gap in the opening during subtle adjustment in the coronal and sagittal planes [10]. Unaddressed disruption of the lateral cortex may result in marked instability at the osteotomy site, loss of angular correction, delayed union or nonunion of the osteotomy, and consequent implant failure [11, 12].

In the previous study, an LHF was seen about 20% compared to non-LHF and various complications of LHF have been reported. In particular, instability was caused by LHF. Such fracture needs to accurately reflect the anatomical and biomechanical characteristics of the various types [13, 14].

Takeuchi et al. developed a new classification for LHFs after opening wedge HTO and hypothesized that bone healing is delayed if a fracture is observed in the distal portion of the tibiofibular joint (type II) owing to its unstable situation [15]. Type II and type III fractures (lateral plateau fracture model) require careful treatment because they are unstable compared with type I fractures (extension of the osteotomy line fracture model).

Although the exact mechanism of fracture remains unclear, an LHF is associated with an increased opening distance of the osteotomy [14]. A previous study involving a large patient series reported that LHFs do not affect bone healing by using internal fixator plates [5]; however, a subdivision of hinge fracture types was not observed. Furthermore, biomechanical, animal, and clinical studies indicated that an LHF after open wedge HTO caused instability that decreased bone healing and led to correction loss and nonunion, especially with unstable plates [9, 16–18]. Currently, computational simulation is widely being used to evaluate the stability of plate design in HTO [19–22]. The advantages of computational simulation for a single subject are that the effects of types of LHF within the same “person” are determined and that the effects of variables such as weight, height, bony geometry, ligament properties, and plate design are excluded [23].

The purpose of the present study was to compare the biomechanical effect of three different Takeuchi classification type LHF HTO models and the HTO model without LHF (non-LHF). The stress on the bone and plate, wedge micromotion, and forces on the anterior cruciate ligament (ACL) and posterior cruciate ligament (PCL) were evaluated to investigate the stress-shielding effect, plate stability, and biomechanical changes, respectively, in the three LHF HTO and non-LHF models. We hypothesized that the LHF type I model (fracture involves an extension of the osteotomy line and is immediately proximal to or within the tibiofibular joint), in which the fracture occurs in the safety zone, would most closely approximate the mechanical effect of non-LHF.

## Methods

### Development of the medial opening wedge HTO model

**Fig. 1:**
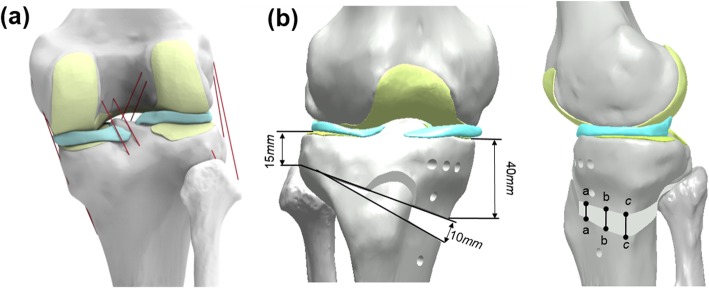
Schematics illustrating intact (**a**) and high tibial osteotomy (HTO) model. Three edges *aa*, *bb*, and *cc* across the opening were defined to calculate changes in length before and after load

An existing, previously validated, finite element (FE) model for the knee joint was used in this study [24–26]. Radiographic data from the knee joint of a 36-year-old male weighing 80 kg and 178 cm in height were acquired using computed tomography (CT) and magnetic resonance imaging (MRI). CT and MRI were performed using a 64-channel CT scanner (Somatom Sensation 64, Siemens Healthcare, Erlangen, Germany) and a 3.0-Tesla MRI scanner (Achieva 3.0T, Philips Healthcare, Netherlands), with slice thicknesses of 0.1 and 0.4 mm, respectively. The process of combining the reconstructed CT and MRI images with the alignment for each model was executed using commercially available software (Rapidformm version 2006, 3D Systems Korea, Seoul, Republic of Korea). The segmented images were exported in stereolithography format and further processed using three-dimensional (3D) modeling software (Mimics version 17.0, Materialise, Leuven, Belgium) to create geometric models (Fig. 1).

The healthy knee model was subsequently used to simulate the medial opening wedge HTO with the distal region of the tibia rotated, while the opening wedge was simulated in the frontal plane to represent valgus correction angles (Fig. 1) [27]. The opening wedge on the medial side was guided by a clinician to simulate HTO. Specifications, including wedge size and correction angle, were described in previous studies [28, 29] and shown in Fig. 1. A 10-mm gap in the opening wedge was simulated by wedge-shaped bone removal from the proximal tibia (Fig. 1). The TomoFix (DePuy Synthes, Warsaw, IN, USA) plate modeled in Unigraphics NX (version 7.0; Siemens PLM Software, Torrance, CA, USA) was virtually implanted into the medial tibia to simulate 88 medial opening wedge HTO fixation.

**Fig. 2:**
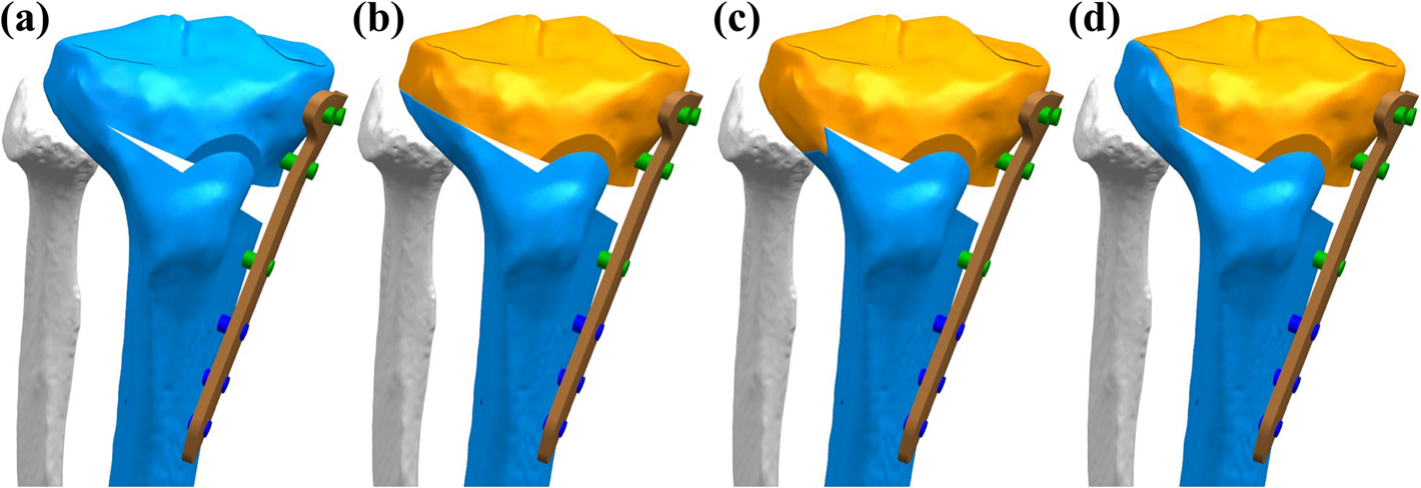
**a** High tibial osteotomy without lateral hinge fracture (non-LHF), **b** Takeuchi classification for lateral hinge fracture (LHF) type I, **c** type II, and **d** type III finite element (FE) models used in this study

An extant study developed the LHF HTO model in accordance with the Takeuchi classification [15]. In the models, fractures around the lateral cortical hinge, including lateral plateau fracture, were classified as three different types (Fig. 2): type I (fracture involves an extension of the osteotomy line and is immediately proximal to or within the tibiofibular joint); type II (fracture reaches the distal portion of the proximal tibiofibular joint); and type III (lateral plateau fracture) [15].

The locking screws of the TomoFix plate were simulated to rigidly bond with the plate screw [19, 30]. To simulate the contact between the bone and screws under loading conditions, a surface-to-surface contact relationship was assumed in the model. Contact pairs were defined between the bone and the screws, with the trailing edge of the screw considered to be the master surface, and the elements of the bone considered to be the slave surface. A friction coefficient of 0.2 was assumed for the contact surface between the bone and screws [31]. The material properties of the titanium alloy used in the HTO plate corresponded to a Young’s modulus of 110 GPa and a Poisson’s ratio of 0.3 [22]. A bone graft was disregarded in the FE analysis to simulate the worst-case scenario for implant loading [19, 30]. The cartilage was modeled as isotropic, and the menisci were modeled as transversely isotropic with linear elastic material properties [32]. To simulate meniscal attachments, each meniscal horn was fixed to the bone using linear spring elements (“SPRINGA” element type), with a total stiffness of 2000 N/mm at each horn [32]. The major ligament models were defined as hyper-elastic rubber-like materials that exhibited nonlinear stress–strain relations [33]. The tibia was not modeled as rigid to evaluate the stress-shielding effect after computational simulation of the HTO [34]. Cortical bone was considered as transversely isotropic (*E*_*x*_ = *E*_*y*_ = 11.5 GPa, *E*_*z*_ = 17 GPa; *G*_xy_ = 3.6 GPa, *G*_xz_ = G_yz_ = 3.3 GPa; *v*_xy_ = 0.51, *v*_xz_ = *v*_yz_ = 0.31 GPa). Cancellous bone was modeled as a linear isotropic material property with *E* = 2.13 GPa and *v* = 0.3 [34]. The femur, however, was modeled as a rigid body [24].

Contacts were established between the femoral cartilage and the menisci, the menisci and the tibial cartilage, and the femoral and tibial cartilages for both the medial and lateral sides, resulting in six contact pairs (Fig. 2). A frictionless surface-to-surface tangential contact with a nonlinear finite sliding property was used to simulate articular surfaces [32, 35–37].

**Fig. 3:**
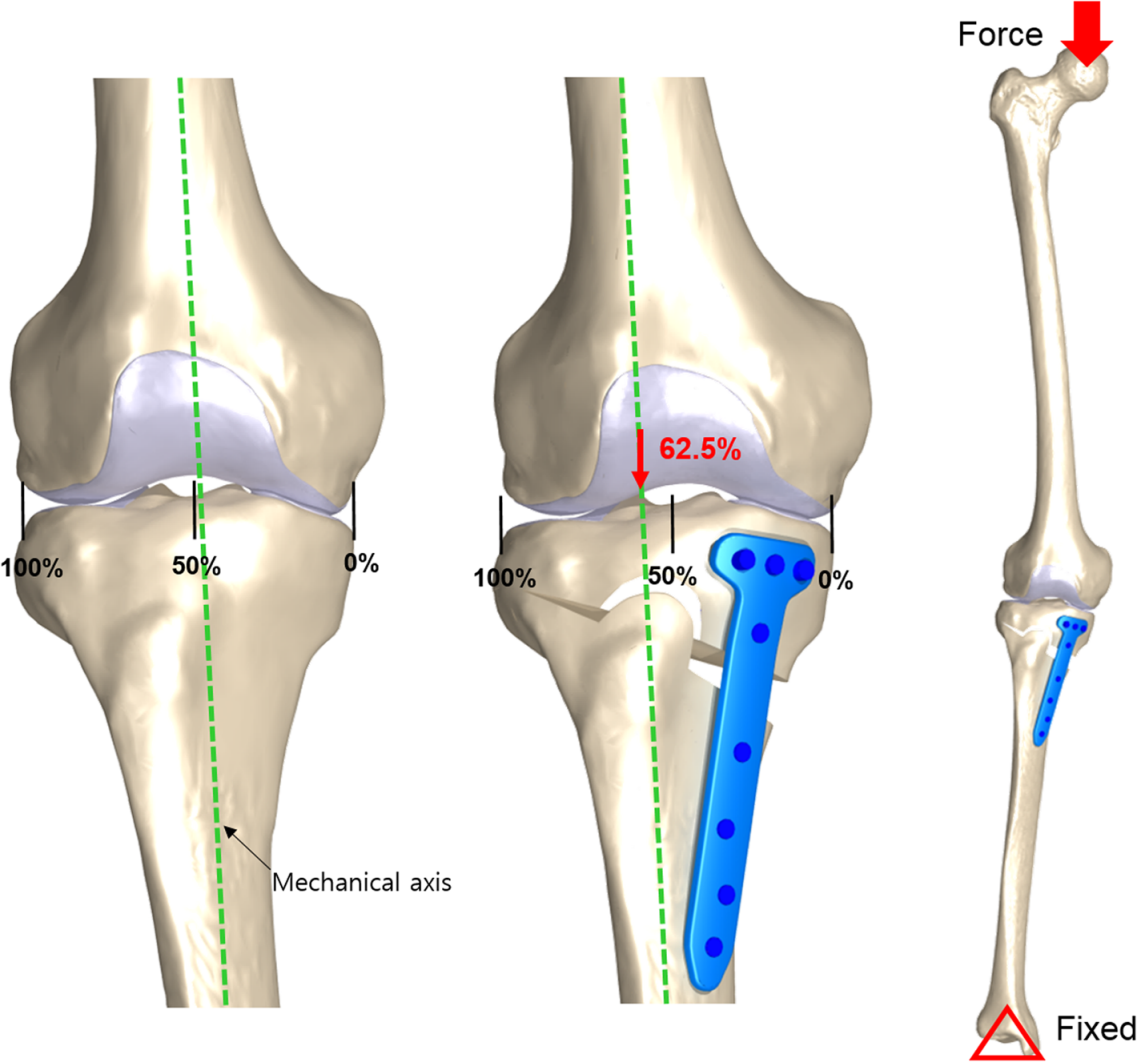
Mechanical axis in the intact (**a**), high tibial osteotomy (HTO) model (**b**), and loading and boundary condition (**c**)

Mesh convergence tests were performed to complete the simulation. Convergence was obtained if the relative change between two adjacent meshes was < 5%. The average element sizes were 0.8 mm for the cartilage and menisci, respectively. Details of the element type and numbers are provided in Table 1 [35]. The medial opening wedge HTO was simulated such that the loading axis of mechanical axis became lateral at 62.5%, as suggested by Fujisawa et al. [26] (Fig. 3).

**Table 1:** Details of element type and numbers used in this study

Set	Element type	Element number
Femur bone	Quad	72,516
Tibia bone	Quad	47,665
Fibula bone	Quad	19,763
Femoral cartilage	Hexa	14,688
Tibial cartilage	Hexa	5556
Medial meniscus	Hexa	2304
Lateral meniscus	Hexa	2430
Anterior cruciate ligament (ACL)	Hexa	2130
Posterior cruciate ligament (PCL)	Hexa	4598
Medial collateral ligament (MCL)	Hexa	4766
Lateral collateral ligament (LCL)	Hexa	1148
Total		177,564

### Loading and boundary conditions

This FE investigation included three types of loading conditions corresponding to the loads used in the experimental study for model validation and model predictions for clinically relevant loading scenarios. With regard to model validation, identical simulated loading protocols were applied in the experiment.

In the first loading condition, 150 N was applied to the tibia with 30 ° flexion in the FE knee joint to measure anterior tibial translation and posterior tibial translation, respectively [38]. Additionally, a second axial load of 1150 N was applied to the model to obtain contact stress to facilitate comparison with the results of a previously published study on knee joint FE analysis [39]. The third loading condition involved a clinically relevant load for each configuration under the same load conditions. A vertical compressive force was applied to the knee joint in full extension. A force of 2500 N, corresponding to 3.1 times the body weight of an individual weighing 80 kg, was applied. This is equivalent to the maximal axial force during the gait cycle [22]. In all tests, the tibia was completely constrained to its distal end [20–22] (Fig. 3).

Three indices were determined to compare the differences in stress and micromotion with the material properties of the HTO plate variations. First, the average stress (von-Mises) on the bone and the plate was investigated. Second, the construct stability for change in the height at edges *aa*, *bb*, and *cc* of the opening was evaluated (Fig. 1). Finally, the force on the ACL and PCL was evaluated to investigate the biomechanical effect on the soft tissue with regard to the LHF HTO.

## Results

### Intact model validation

For FE model validation, the results from the experiment were compared with the FE subject. Under the loading condition with 30° flexion, anterior tibial translation was 2.83 mm in the experiment and 2.54 mm in the FE model, and posterior tibial translation was 2.12 mm in the experiment and 2.18 mm in the FE model; thus, good agreement between the experimental results and the FE model was observed (Table 2) [38].

**Table 2:** Comparison of anterior and posterior tibial translation for validation of the model under the 30° flexion loading condition

	Previous study [38]	The present study
Anterior tibial translation (mm)	2.83	2.54
Posterior tibial translation (mm)	2.12	2.18

Additionally, the results were also compared with previous FE results for model validation. Maximum contact stresses corresponding to 3.1 MPa and 1.53 MPa were observed on the medial and lateral menisci, respectively, under an axial load of 1150 N. Both were within 4% of the contact stresses, corresponding to 2.9 MPa and 1.45 MPa, respectively, as reported in a previous study [39]. The minor differences were potentially due to geometric variations such as the thickness of the cartilage and meniscus in each study. However, overall, considerable consistency between the results of validation and the literature confirmed the ability of the FE model to produce reasonable results.

### Stress on the bone and plate, forces on the ACL and PCL, and micromotion of the wedge in the LHF HTO model

**Fig. 4:**
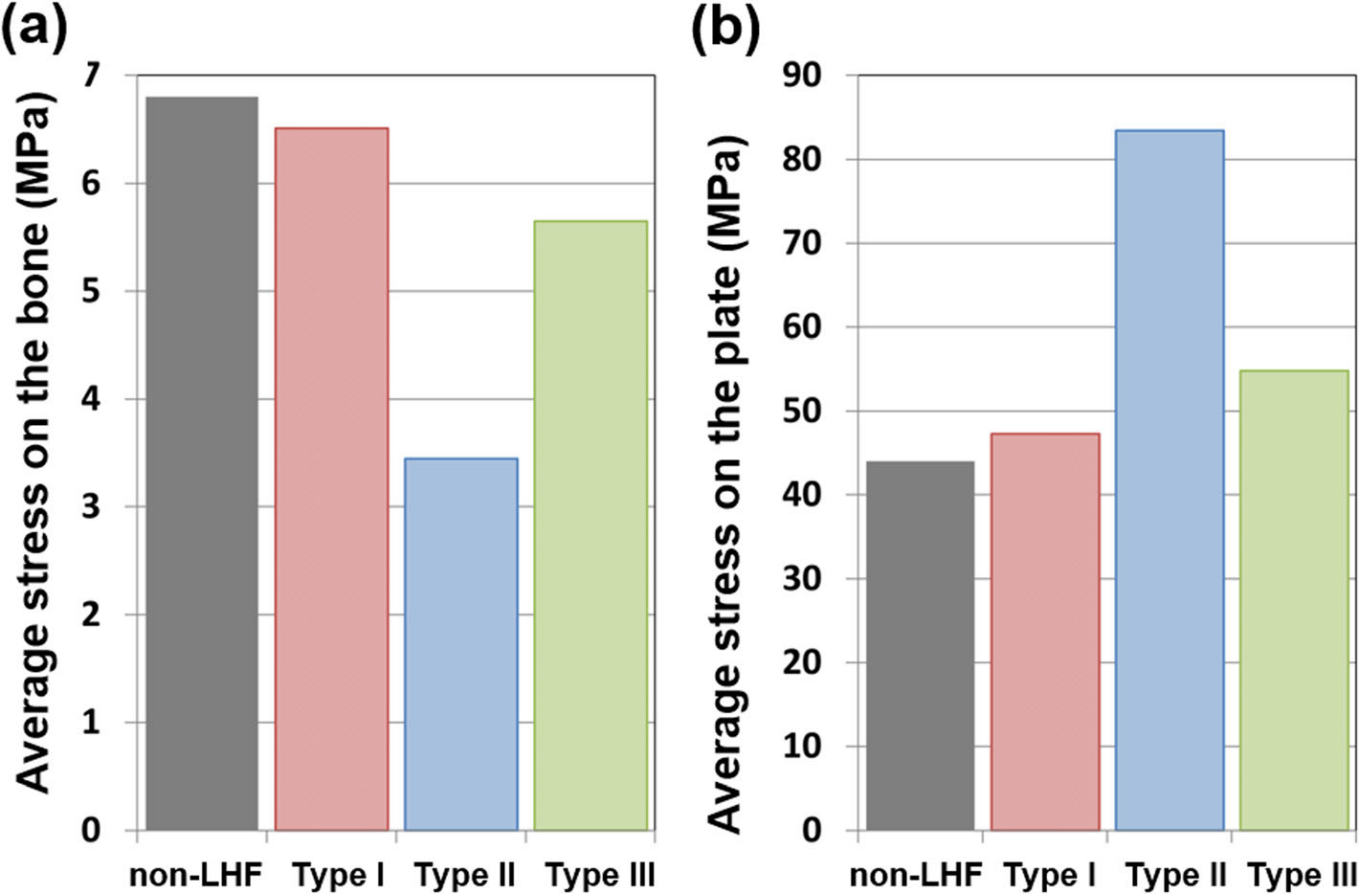
Comparison of bone and plate stress in high tibial osteotomy without lateral hinge fracture (non-LHF) and lateral hinge fracture (LHF) models after weight bearing

**Fig. 5:**
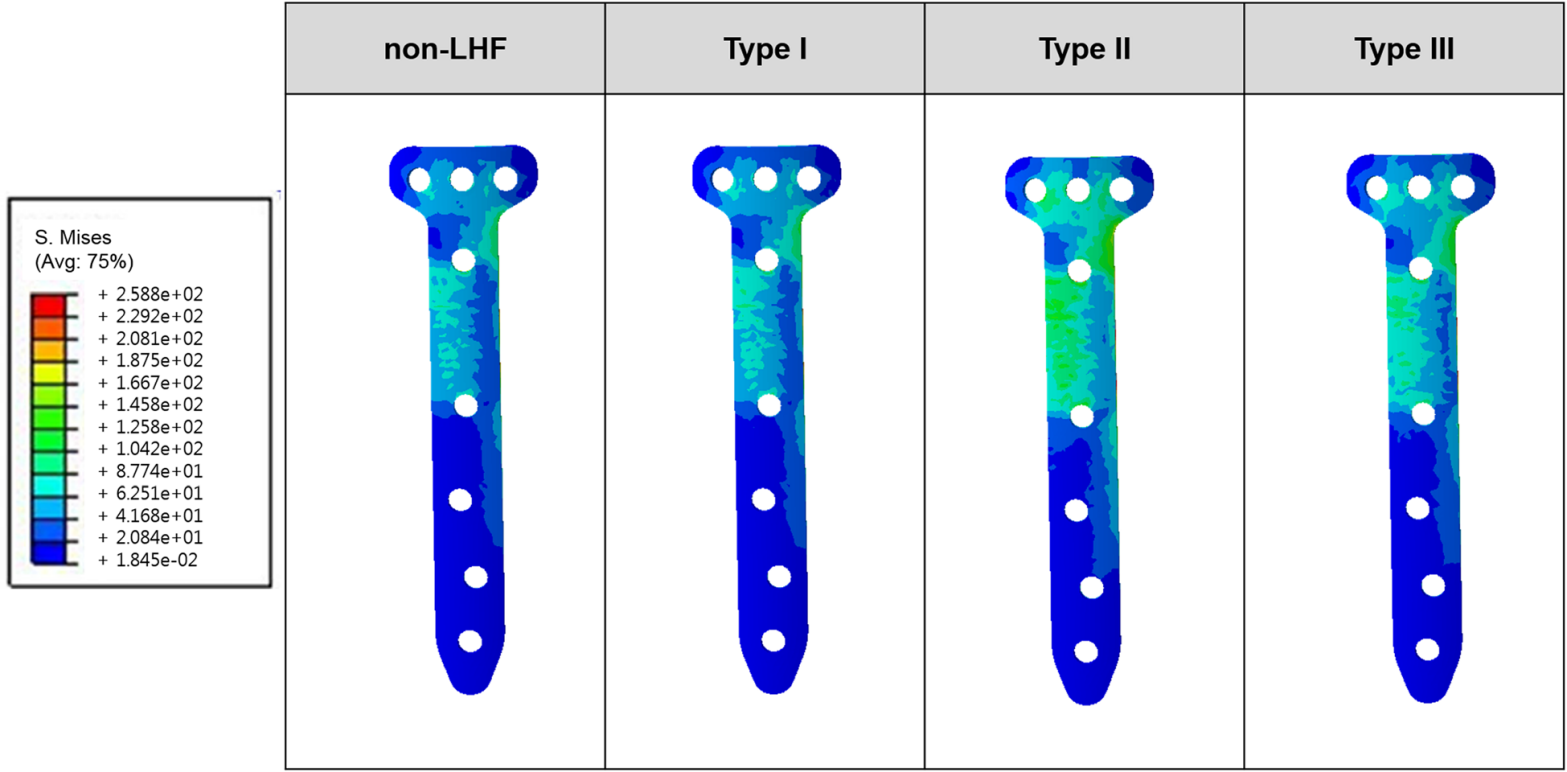
Stress distribution of plate in the lateral hinge fracture (LHF) high tibial osteotomy (HTO) without LHF (non-LHF) models

**Fig. 6:**
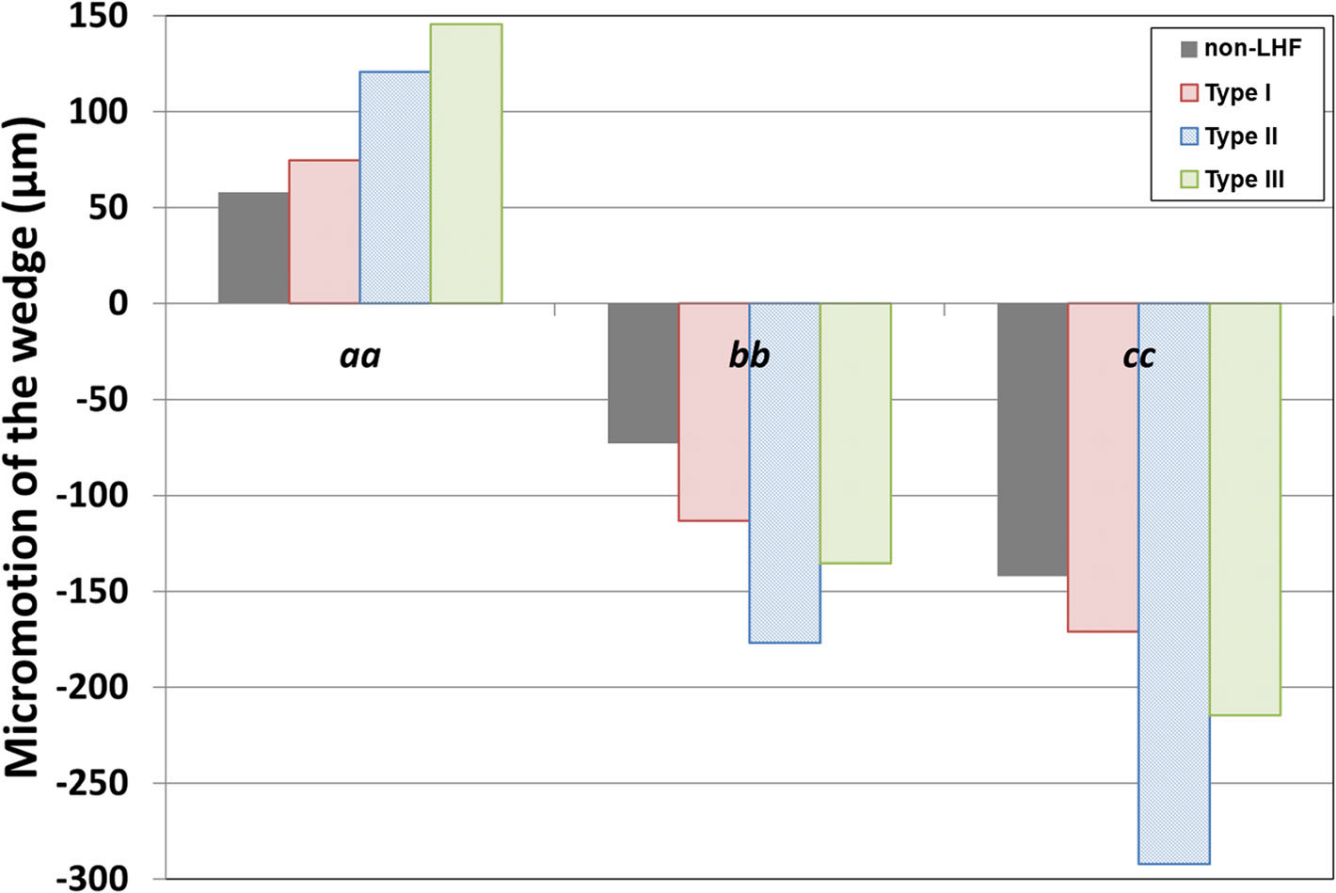
Comparison of wedge micromotion in high tibial osteotomy without lateral hinge fracture (non-LHF) and lateral hinge fracture (LHF) models after weight bearing

**Fig. 7:**
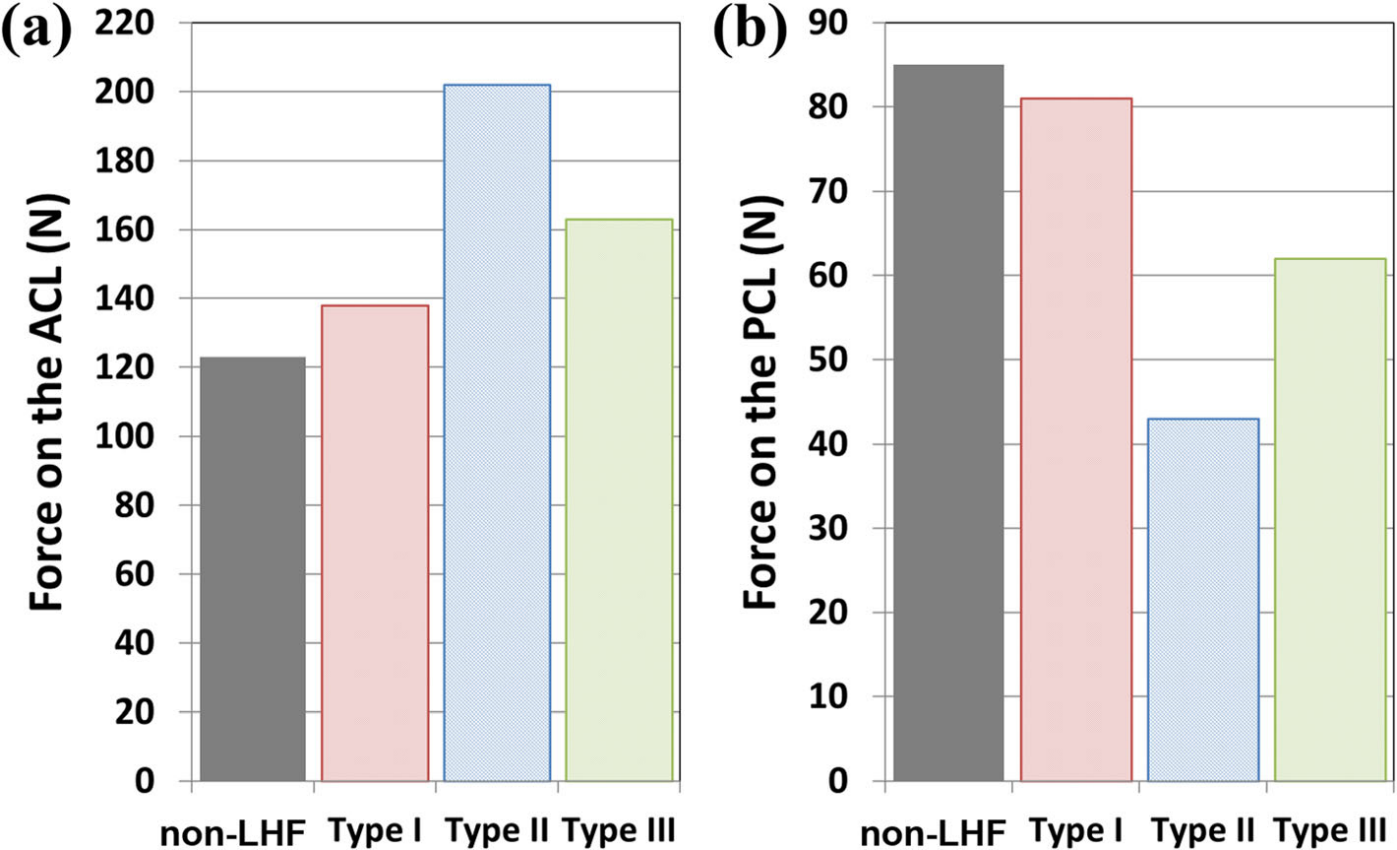
Comparison of anterior cruciate ligament (ACL) and posterior cruciate ligament (PCL) forces in high tibial osteotomy without lateral hinge fracture (non-LHF) and lateral hinge fracture (LHF) models after weight bearing

The bone and plate stresses in the LHF HTO and non-LHF models are shown in Fig. 4. The greatest bone stress and the lowest plate stress were observed in the non-LHF model. Stresses on the bone were 7.1 MPa, 6.8 MPa, 3.6 MPa, and 5.9 MPa in the non-LHF, type I, type II, and type III LHF HTO models, respectively. An opposite trend was observed for plate stress. Stresses on the plate were 52.4 MPa, 79.8 MPa, 45.2 MPa, and 42.1 MPa in type III, type II, type I LHF HTO, and non-LHF models, respectively. Type I, type III, and type II models exhibited 7%, 24%, and 90% higher plate stress, respectively, compared with that of the non-LHF model. The plate stress distribution in the LHF HTO and non-LHF models is shown in Fig. 5. Stress concentration on the wedge region was found in the type II model compared with the non-LHF model. Wedge micromotion in the LHF HTO and non-LHF models is shown in Fig. 6. The largest micromotions among all models were observed in the region at edge *cc*. Additionally, tension and compression were exerted at edges *aa*, *bb*, and *cc* in all models. The lowest micromotions were observed in the non-LHF model. Forces on the ACL and PCL in the LHF HTO and non-LHF models are shown in Fig. 7. Forces on the ACL and PCL in the type LHF model were similar to those in the non-LHF model. However, increased force on the ACL and decreased force on the PCL were observed in the type II and type III models compared with those in the non-LHF model. In particular, ACL force increased by 64% and PCL force decreased by 49%, respectively, in the type II model compared with those in the non-LHF model.

## Discussion

The most important finding of this study was that a stress-shielding effect was observed due to the increased plate stress and reduced bone stress in Takeuchi type II and type III fractures, leading to delayed union. Additionally, stability decreased in type II and type III fractures due to increased wedge micromotion. However, a similar biomechanical effect was observed with non-LHF in type I fractures, in which the LHF was in the safety zone.

A previous study reported that a lateral cortex fracture, as a complication of opening medial wedge HTO using a full bony wedge in the osteotomy gap, led to the displacement of the osteotomy and recurrent varus malalignment before osteotomy union in 12% of patients [40]. A complete fracture increased micromotion as well as shear and tensile forces on the construct, which, in turn, decreased the threshold for implant failure. Other studies demonstrated an approximately 12-fold increase in the rate of nonunion and collapses in cases without preserving the integrity of the cortical hinge [41]. Most studies have only reported intraoperative fractures [10]. Reports of cortical hinge fracture after 6 weeks (postsurgical lateral cortical fracture) are rare [10]. A previous study reported intraoperative lateral cortex fractures in 3% and an additional 6% of patients during follow-ups [42]. A possible mechanism was that the apex of the locking screw generated a new hinge point, with a maximum load on the apex leading to a fracture in the tibia during partial weight bearing [42]. The lateral hinge is important for primary stability [43]. Fracture of the lateral cortex causes considerable reduction in axial and rotational stiffness, as well as an increase in micromotion at the osteotomy site [11]. This may lead to a loss of angular correction, a delayed union, or even nonunion of the osteotomy [12]. However, previous studies did not evaluate the biomechanical mechanism with LHF in the opening wedge HTO. The main intention of the present study was to evaluate the effect of the Takeuchi classification in LHF and confirm the biomechanical mechanism.

To perform testing in the present study, we developed a 3D nonlinear FE model of the knee joint with bony structures and soft tissues, including ligaments, menisci, and articular cartilage. We used the healthy knee to investigate biomechanical effect of the LHF type. The intact knee model involved a series of rigorous validation steps. The results correlated well with previous experiments, an experiment using the same subject, and previous FE studies. Therefore, we believe that the LHF HTO models used in this study and the subsequent analyses are reasonable. The study introduced the following three factors concerned with stabilization of the medial opening: effective load-sharing mechanism at the bone and plate; micromotion between 100 and 200 μm, with maintained stability for callus formation and mineralization; and similar biomechanical effects between the LHF HTO and non-LHF models.

The results of our study indicated that the stress-shield effect―not load sharing―was observed in the type II and type III models. Additionally, similar bone and plate stress was demonstrated in the type I and non-LHF models. Thus, more load sharing existed in type I than those in types II and III. As indicated by Takeuchi et al., the dense and solid connective tissues in the proximal tibiofibular joint act as an anatomical advantage for healing in type I fractures [15]. However, in type II fractures, the energy of opening the osteotomy site is accumulated in the fibula and corresponds to rotation energy when a fracture line is attained by the lateral cortex distally to the proximal tibiofibular joint [15]. External rotation of the two fragments was caused by the energy and led to delayed union or nonunion of the osteotomy site and correction loss [15]. In the type III fracture, a serious complication existed because the articular surface of the lateral compartment was injured. Regarding the alignment from varus to valgus, the weight-bearing line shifted from the medial plateau of the tibia to the lateral plateau [15]. Type II and type III models cannot support loading compared with the type I model in the proximal tibiofibular joint. It led to instability of the knee joint that caused an increase in plate stress and, furthermore, led to a decrease in bone stress and caused a stress-shielding effect.

The results were also obtained in wedge micromotion, which was high in type II and type III models due to instability. A previous study reported that lateral loading of the tibia produced increased weight on the fibula, which indicated an important role of the open wedge HTO that potentially involves supporting the lateral tibial plateau [44]. In the type II model, the fracture is not loaded as it exits below the proximal tibiofibular joint, which is a potential reason for the instability. Additionally, type III fractures are unstable because the proximal fragment is only supported by the HTO plate; hence, stress shielding and instability are potentially observed in the type II and type III models. Fixation stability is important if LHFs occur, as indicated by Agneskirchner et al., who suggested the superior stability of the TomoFix plate compared with those of other less-rigid plates in the presence of LHFs produced during the biomechanical testing of open wedge HTOs in artificial bones [8]. Clinical studies have observed that more complications, such as loss of correction, are observed in patients with LHFs after open wedge HTO fixed with small spacer plates [5, 12, 14–18]. An unstable osteotomy or fixation construct necessitates the adaptation of the weight-bearing protocol and postpones full weight bearing. We previously suggested the maximum values (> 100 μm, < 200 μm) for allowable micromotion movement for bone union [45]. Theoretically, however, the trade-off between stability and interfacial micromotion remains unclear [22]. The adequate micromotion of fracture interfaces can enhance callus formation [46, 47]. Type II and type III models demonstrated micromotion in excess of 200 μm and indicated reduced stability and potential delay union.

An interesting finding was related to the forces on the ACL and PCL. A previous meta-analysis indicated that posterior tibial slope increased after the open wedge HTO and decreased after closed wedge HTO when the results of a variety of measurement methods were examined [48]. Generally, a low hinge position during the medial open wedge HTO resulted in a significantly greater increase in the posterior tibial slope compared with the standard hinge position [49]. Our results indicated that type II and type III fractures increased the posterior tibial slope in the weight-bearing condition because only the TomoFix plate supported the load. Theoretically, an increased posterior tibial slope induces higher anterior tibial translation under the tibiofemoral compression force. Increased posterior tibial slope and anterior tibial translation leads to higher ACL force accompanied by a lower PCL force. The results indicated an increase in ACL force and a decrease in PCL force in the type II and type III LHF models compared with the type I and non-LHF models. In other words, type II and type III LHF models indicated an increase in posterior tibial slope. It was also confirmed by wedge micromotion evaluated in this study. Micromotion at the posterior region in the wedge increased and led to increased posterior tibial slope. The ACL force increased when the posterior tibial slope increased and indicated good agreement with the results of the previous study in which PCL force decreased [50–52].

As previously mentioned, type I fractures are relatively stable because the soft tissue near the proximal tibiofibular joint area is dense and solid. Additionally, the load from the fibula to the fracture plane under a weight-bearing condition may enhance fracture healing. Therefore, type I fractures exhibit a biomechanical effect similar to non-LHF.

It is important to highlight the three main advantages of the present study. First, only the tibia was modeled in previous studies that simulated an HTO plate [19–22, 30]. However, a proper knee joint, including soft tissue, was modeled in the present study. Second, the initial FE model was validated before simulation in this study, whereas the FE model was not validated in previous studies [19–22, 30]. Third, the bonding condition was applied between the bone and screw, and this was assumed to be a bone union, although it does not correspond to a realistic simulation. Therefore, a contact condition between the bone and screw was applied [19–22, 30].

Although the present study provides valuable insights into the biomechanical roles of LHF HTO, there were limitations. First, simulations were performed only under static conditions because the ideal dynamic motion of the joint was too prohibitive in terms of computing resources and time. In future studies, we may explore a more suitable representation of the joint as well as an analysis of the system under cyclic loading. Second, material properties used in the computational model were referred to in extant studies.

## Conclusions

This study demonstrated that Takeuchi type II and type III LHF models provide unstable structures compared with those of the type I and non-LHF models. Takeuchi type II and type III LHF models potentially lead to a delay in union due to increased plate stress and reduced bone stress compared with type I and non-LHF models. Our results suggest that HTO should be performed while undertaking a medial opening wedge HTO to avoid the complication of an LHF in type II and type III models.

## Data Availability

Not applicable

## Abbreviations

3D: Three-dimensional
ACL: Anterior cruciate ligament
CT: Computed tomography
FE: Finite element
HTO: High tibial osteotomy
LHF: Lateral hinge fracture
MRI: Magnetic resonance imaging
non-LHF: HTO model without LHF
PCL: Posterior cruciate ligament
